# Supplementary material for: Hedgehogs, Squirrels, and Blackbirds as Sentinel Hosts for Active Surveillance of Borrelia miyamotoi and Borrelia burgdorferi Complex in Urban and Rural Environments
Source: Microorganisms. 2020 Nov 30;8(12):1908. doi: 10.3390/microorganisms8121908 (PMC7760222; doi:10.3390/microorganisms8121908)
Supplement: Supplementary file 1 [file microorganisms-08-01908-s001.zip › Supplementary data manuscript FINAL/Table S1 Grade of autolysis.pdf]

Table S1: Grade of autolysis

Scoring of the grade of the carcass autolysis:	
0	An alive animal, which was immediately before necropsy euthanized
1A	FRESHLY DEAD – approximately 1-3 hours from the death in fresh weather. The carcass is intact and does not smell rotten, the fur is difficult to pull out and skin is intact with minimal or no sloughing observed. The animal is not bloated.
1B	LESS FRESHLY DEAD - approximately 1-3 hours from the death in warmer weather or more than 4 hours in fresh weather. The carcass seems intact and start to smell rotten, the fur is difficult to pull out and skin is intact with minimal or no sloughing observed. The animal or organs are starting bloated.
2	MODERATELY DECOMPOSED - approximately 4-6 hours from the death in warmer weather or more than half of the day in fresh weather. There is moderate gas distension. The fur appears to be in the early stages of sloughing. The smell of decomposition is clearly discernible if the carcass is approached.
3	ADVANCED DECOMPOSITION. The carcass is much distended with gas and tissue is sloughing away from the face. The cadaver is soft, having already passed the bloated stage. There is a lot of sloughing over the whole body. The smell of decomposition is typically strong.
4	MUMMIFIED - postmortem deterioration has advanced to the point that the carcass has become a flattened and desiccated shell. At this stage, limbs and internal organs are often missing or unrecognizable. At a distance the smell of decomposition may be surprisingly mild for mummified carcasses as the process of decomposition is nearing completion, with only the toughest tissue parts remaining: The bones and hide. The skeleton can be with bits of tissue.
