# Supplementary material for: Hedgehogs, Squirrels, and Blackbirds as Sentinel Hosts for Active Surveillance of Borrelia miyamotoi and Borrelia burgdorferi Complex in Urban and Rural Environments
Source: Microorganisms. 2020 Nov 30;8(12):1908. doi: 10.3390/microorganisms8121908 (PMC7760222; doi:10.3390/microorganisms8121908)
Supplement: Supplementary file 1 [file microorganisms-08-01908-s001.zip › Supplementary data manuscript FINAL/Table S2 PCR primers and protocols.pdf]

Table S2: PCR primers and protocols

multiplex real-time PCR

**OspA(Outer membrane Protein A)**	± 139bp	reference: Heylen, D.J.A.; Tijsse, E.; Fonville, M.; Matthysen, E.; Sprong, H. Transmission dynamics of *Borrelia burgdorferi* s.l. in a bird tick community. *Environ. Microbiol* **2013**, *15*, 663–673.
F-primer :	5'-AAT ATT TAT TGG GAA TAG GTC TAA-3'	
R-primer:	5'-CTT TGT CTT TTT CTT TRC TTA CA-3'	
Probe (Atto520):	5'-**Atto520**-AAG CAA AAT GTT AGC AGC CTT GA-**BHQ1**-3'	
**FlaB(Flagelin B)**	± 89bp	reference: Heylen, D.J.A.; Tijsse, E.; Fonville, M.; Matthysen, E.; Sprong, H. Transmission dynamics of *Borrelia burgdorferi* s.l. in a bird tick community. *Environ. Microbiol* **2013**, *15*, 663–673.
F-primer :	5'-CAG AIA GAG GTT CTA TAC AIA TTG AIA TAG A-3'	
R-primer1:	5'-GTG CAT TTG GTT AIA TTG CGC-3'	
R-primer2:	5'-GTG CAT TTG GTT AIA TTG TGC-3'	
Probe (Atto425):	5'-**Atto425**-CAA CTI ACA GAI GAA A**X**T AAI AGA ATT GCT GAI CA-**Pho**-3'	
	**X** = BHQ-1-dT	
**FlaB *B. miyamotoi***	± 155bp	reference: Hovius, J.W.R.; de Wever, B.; Sohne, M.; Brouwer, M.C.; Coumou, J.; Wagemakers, A.; Oei, A.; Knol, H.; Narasimhan, S.; Hodiamont, C.J.; Jahfari, S.; Pals, S.T.; Horlings, H.M.; Fikrig, E.; Sprong, H.; van Oers, M.H.J. A case of meningoencephalitis by the relapsing fever spirochaete *Borrelia miyamotoi* in Europe. *Lancet* **2013**, *382*, 658
F-primer :	5'-AGA AGG TGC TCA AGC AG-3'	
R-primer:	5'-TCG ATC TTT GAA AGT GAC ATA T-3'	
Probe (Atto647N):	5'-**ATTO647N**-AGC ACA ACA GGA GGG AGT TCA AGC-**BHQ2**-3'	

IGS conventional PCR

**IGS primers**	± 390-420bp	reference: Heylen, D.J.A.; Tijsse, E.; Fonville, M.; Matthysen, E.; Sprong, H. Transmission dynamics of *Borrelia burgdorferi* s.l. in a bird tick community. *Environ. Microbiol* **2013**, *15*, 663–673.
F-primer :	5'-GAGTTCGCGGGAGAGTAGGTTATTGCC-3'	
R-primer:	5'-TCAGGGTACTTAGATGGTTCACTTCC-3'	
**IGS sequencing - short primers**		
F-primer :	5'-GCGGGAGAGGTTATTGCC-3'	
R-primer:	5'-GTACTTAGATGGTTCACTTCC-3'	

**IGS PCR protocol**	temperature (°C)	Time (hh:mm:ss)	Cycles
Taq activation	94	0:15:00	1
Denaturation	94	0:00:20	10
Annealing	70	0:00:30	(lowering 1˚C per cycle)
Elongation	72	0:00:30	
Denaturation	94	0:00:20	40
Annealing	60	0:00:30	
Elongation	72	0:00:30	
Extension	72	0:07:00	1
Hold	12	forever	

FlaB conventional PCR

**FlaB primers**	± 497 bp	reference: Clark, K; Hendricks, A.; Burge, D. Molecular identification and analysis of *Borrelia burgdorferi* sensu lato in lizards in the southeastern United States. *Appl Environ Microbiol*. **2005**, *71*, 2616-2625
F-primer :	5'-AARGAATTGGCAGTTCAATC-3'	
R-primer:	5'-GCATTTTCWATTTTAGCAAGTGATG-3'	

**FlaB PCR protocol**	temperature (°C)	Time (hh:mm:ss)	Cycles
Taq activation	96	0:05:00	1
Denaturation	95	0:00:30	30
Annealing	52	0:00:30	
Elongation	72	0:01:00	
Extension	72	0:10:00	1
Hold	12	forever	

***ospC* conventional nested PCR**

***ospC* outer primers**	± 657 bp	reference: Bunikis, J.; Garpmo, U.; Tsao, J.; Berglund, J.; Fish, D.; Barbour, AG. Sequence typing reveals extensive strain diversity of the Lyme borreliosis agents *Borrelia burgdorferi* in North America and *Borrelia afzelii* in Europe. *Microbiology* **2004**, *150*, 1741–1755
F-primer :	5'-ATGAAAAAGAATACATTAAGTGC-3'	
R-primer:	5'-ATTAATCTTATAATATTGATTTTAATTAAGG-3'	
***ospC* inner primers**	± 617 bp	
F-primer :	5'-TATTAATGACTTTATTTTTATTTATATCT-3'	
R-primer:	5'-TTGATTTTAATTAAGGTTTTTTTGG-3'	

***ospC* 1st PCR protocol**	temperature (°C)	Time (hh:mm:ss)	Cycles
Taq activation	95	0:05:00	1
Denaturation	94	0:00:30	35
Annealing	52	0:00:30	
Elongation	74	0:00:30	
Extension	72	0:10:00	1
Hold	12	forever	
***ospC* nested PCR protocol**	temperature (°C)	Time (hh:mm:ss)	Cycles
Taq activation	95	0:05:00	1
Denaturation	94	0:00:30	25
Annealing	52	0:00:30	
Elongation	74	0:00:30	
Extension	72	0:10:00	1
Hold	12	forever	

***ospA* species specific PCR**		reference: Demaerschalck, I.; Ben Messaoud, A.; De Kesel, M.; Hoyois, B.; Lobet, Y.; Hoet, P.; Bigaignon, G.; Bollen, A.; Godfroid, E. Simultaneous presence of different *Borrelia burgdorferi* genospecies in biological fluids of Lyme disease patients. *J Clin Microbiol*. **1995**, *33*, 602–608.
**GI primers set**	± 543 bp	
F-primer :	5’-AACAAAGACGGCAAGTACGATCTAATT-3’	
R-primer:	5’-TTACAGTAATTGTTAAAGTTGAAGTGCC-3’	
**GII primers set**	± 344 bp	
F-primer :	5’-TGATAAAAACAACGGTTCTGGAAC-3’	
R-primer:	5’-GTAACTTTCAATGTTGTTTTGCCG-3’	
**GIII primers set**	± 189 bp	
F-primer :	5’-TAAAGACAAAACATCAACAGATGAAATG-3’	
R-primer:	5’-TTCCAATGTTACTTTATCATTAGCTACTT-3’	

***ospA* PCR protocol**	temperature (°C)	Time (hh:mm:ss)	Cycles
Taq activation	95	0:05:00	1
Denaturation	95	0:00:30	35
Annealing	55	0:00:30	
Elongation	72	0:01:00	
Extension	72	0:10:00	1
Hold	12	forever	
