# Supplementary material for: Hedgehogs, Squirrels, and Blackbirds as Sentinel Hosts for Active Surveillance of Borrelia miyamotoi and Borrelia burgdorferi Complex in Urban and Rural Environments
Source: Microorganisms. 2020 Nov 30;8(12):1908. doi: 10.3390/microorganisms8121908 (PMC7760222; doi:10.3390/microorganisms8121908)
Supplement: Supplementary file 1 [file microorganisms-08-01908-s001.zip › Supplementary data manuscript FINAL/Table S3 Samples database.pdf]

Table S3: Samples database

EE = *Erinaceus europaeus* , ER = *Erinaceus roumanicus* , SV = *Sciurus vulgaris* , TM = *Turdus merula*
Tissue code: 1 = ear, 2 = skin, 3 = muscle, 4 = blood, 5 = lungs, 6 = liver, 7 = spleen, 8 = urinary bladder, 9 = kidney, 10 = brain
Borrelia burgdorferi s.l. genospecies code: 1 = *B. afzelii* , 2 = *B. garinii* , 3 = *B. bavariensis* , 4 = *B. burgdorferi* s.s., 5 = *B. spielmanii* , 6 = *B. valaisiana* , 7 = *B. carolinensis* /*bissettii* , NI = not identified
ARS = Animal rescue station (no data about place of finding)

sample		Cp values from real-time PCR											
animal	tissue	*Borrelia* FlaB	*Borrelia ospA*	*Borrelia miyamotoi* FlaB	*Borrelia burgdorferi* s.l. genospecies identification	sex (F/M/X)	age (Juv/Ad)	weight (g)	foot lenght (mm)	date of finding	latitude	longitude	grade of autolysis
1 ER	1	-	-	-	-	M	Ad	707	53	21.03.2017	49°36'48''	17°15'1.155''	2
1 ER	3	-	-	-	-								
1 ER	4	-	-	-	-								
1 ER	5	-	-	-	-								
1 ER	6	-	-	-	-								
1 ER	7	-	-	-	-								
1 ER	8	-	-	-	-								
1 ER	9	-	-	-	-								
1 ER	10	-	-	-	-								
2 ER	1	-	36,8	-	1	M	Ad	509	38	07.04.2017	49°13'01.6"	16°34'32.8"	2
2 ER	3	-	36,72	-	1, 2, 4								
2 ER	8	-	36,17	-	2, 4								
3 ER	1	-	-	-	-	M	Ad	725	39	18.04.2017	49°2'4.273"	16°55'30.030"	1B
3 ER	3	-	-	-	-								
3 ER	8	-	-	-	-								
4 ER	1	-	36,6	-	NI	M	Ad	814	40	16.04.2017	49°10'38.519"	16°29'15.258"	1A
4 ER	3	-	-	-	-								
4 ER	4	-	-	-	-								
4 ER	5	-	-	-	-								
4 ER	6	-	-	-	-								
4 ER	7	-	-	-	-								
4 ER	8	-	-	-	-								
4 ER	9	-	-	-	-								
4 ER	10	-	-	-	-								
5ER	1	-	35,35	-	NI	F	Ad	646	39	23.04.2017	49°13'39.250"	16°37'52.310"	1A
5ER	3	-	-	-									
5ER	4	-	-	-									
5ER	5	-	-	-									
5ER	6	-	-	-									
5ER	7	-	-	-									
5ER	8	-	-	-									
5ER	9	-	-	-									
5ER	10	-	-	-									
6 TM	2	36,07	32,73	-	6	F	Ad	81	37	26.04.2017	49°21'21.237"	16°47'0.389"	1A
6 TM	3	-	-	-	-								
6 TM	4	-	-	-	-								
6 TM	6	-	-	-	-								
6 TM	10	-	-	-	-								
7 EE	1	35,59	32,04	-	1, 7	M	Ad	796	40,2	27.04.2017	49°2'26.758"	16°55'5.099"	1A
7 EE	3	26,82	23,25	-	2, 5								
7 EE	4	35,97	33,85	-	4, 7								
7 EE	5	-	-	-	-								
7 EE	6	34,05	31,07	-	1								
7 EE	7	-	-	-	-								
7 EE	8	27,15	23,46	-	1, 2								
7 EE	9	32,99	29,73	-	1								
7 EE	10	37,61	35,47	-	1								
8 ER	1	-	-	-	-	M	Ad	613	38,5	01.05.2017	49°8'44.445"	16°59'29.499"	1A
8 ER	3	-	-	-	-								
8 ER	4	-	-	-	-								
8 ER	5	-	-	-	-								
8 ER	6	-	-	-	-								
8 ER	7	-	-	-	-								
8 ER	8	36,24	33,36	-	1, 2								
8 ER	9	-	-	-	-								
8 ER	10	-	-	-	-								
9 ER	2	-	-	-	-	M	Ad	678	42	14.04.2017	49°2'2.365"	16°18'22.477"	2
9 ER	3	-	-	-	-								
10 TM	2	36,97	33,47	-	6	F	Ad	110	34	04.06.2017	49°11'38.457"	15°49'45.003"	1A
10 TM	3	-	-	-	-								
10 TM	4	-	-	-	-								
10 TM	6	-	-	-	-								
10 TM	10	-	-	-	-								
11 TM	2	-	-	-	-	F	Ad	72	35	24.06.2017	49°9'18.875"	16°20'58.241"	2
11 TM	3	-	-	-	-								
11 TM	6	-	-	-	-								
11 TM	10	38,6	34,79	-	2								
12 SV	1	38,96	37,58	-	2	M	Ad	332	40	04.05.2017	49°19'12.964"	16°20'41.851"	1A
12 SV	3	-	-	-	-								
12 SV	4	-	-	-	-								
12 SV	5	-	-	-	-								
12 SV	6	-	-	-	-								
12 SV	7	-	-	-	-								
12 SV	8	38,15	-	-	1, 2								
12 SV	9	-	-	-	-								
12 SV	10	-	-	-	-								
13 TM	2	-	-	-	-	F	Ad	78	32	23.05.2017	49°11'55.786"	16°36'29.479"	1A
13 TM	3	-	-	-	-								
13 TM	4	-	-	-	-								
13 TM	6	-	-	-	-								
13 TM	10	-	-	-	-								
14 ER	1	-	-	-		F	Ad	675	36	16.06.2017	49°13'3.392"	16°35'52.181"	1B
14 ER	3	39,58	35,47	-	1, 4								
14 ER	4	-	-	-									
14 ER	5	-	-	-									
14 ER	6	-	-	-									
14 ER	7	-	-	-									
14 ER	8	-	39,44	-	4								
14 ER	9	-	-	-									
14 ER	10	-	-	-									
15 TM	2	-	-	-	-	F	Ad	78	36	02.07.2017	49°13'3.392"	16°35'52.181"	2
15 TM	3	-	-	-	-								
15 TM	4	-	-	-	-								
15 TM	6	-	-	-	-								
15 TM	10	-	-	-	-								
16 TM	2	-	-	-	-	F	Ad	91	33	10.06.2017	49°12'21.629"	16°29'29.433"	2
16 TM	3	-	-	-	-								
16 TM	4	-	-	-	-								
16 TM	6	-	-	-	-								

17 EE	1	38,11	34,21	-	1	F	Ad	657	37	23.07.2017	49°25'23.087"	16°21'8.248"	1A
17 EE	3	-	-	-	-								
17 EE	4	-	-	-	-								
17 EE	5	-	-	-	-								
17 EE	6	-	-	-	-								
17 EE	7	-	-	-	-								
17 EE	8	-	-	-	-								
17 EE	9	-	-	-	-								
17 EE	10	-	-	-	-								
18 TM	2	-	36,12	-	2	F	Ad	98	35	24.07.2017	49°25'23.087"	16°21'8.248"	1A
18 TM	3	-	-	-									
18 TM	4	-	-	-									
18 TM	6	-	36,13	-	NI								
18 TM	10	-	-	-									
19 TM	2	-	-	-	-	F	Ad	80	34	04.07.2017	49°12'48.180"	16°35'33.407"	1B
19 TM	3	-	-	-	-								
19 TM	4	-	-	-	-								
19 TM	6	-	-	-	-								
19 TM	10	-	-	-	-								
20 TM	2	37,54	34,32	-	2	F	Ad	101	31	11.05.2017	49°17'15.635"	16°27'24.082"	1B
20 TM	3	-	-	-	-								
20 TM	4	-	-	-	-								
20 TM	6	-	-	-	-								
20 TM	10	-	-	-	-								
21 SV	1	-	-	-	-	F	Ad	384	52	28.07.2017	49°11'44.750"	16°31'59.981"	1A
21 SV	3	-	-	-	-								
21 SV	4	-	-	-	-								
21 SV	5	-	-	-	-								
21 SV	6	-	-	-	-								
21 SV	7	-	-	-	-								
21 SV	8	-	35,8	-	NI								
21 SV	9	-	-	-	-								
21 SV	10	-	-	-	-								
22 SV	1	35,93	33,93	-	4	M	Ad	312	49	22.03.2016	49°16'44.494"	16°27'22.537"	1A
22 SV	3	40,48	38,64	-	NI								
22 SV	4	-	-	-	-								
22 SV	5	-	-	-	-								
22 SV	6	-	-	-	-								
22 SV	7	-	-	-	-								
22 SV	8	-	-	-	-								
22 SV	9	-	-	-	-								
23 SV	1	33,68	31,64	-	4	M	Ad	298	51	26.02.2016	49°17'15.635"	16°27'24.082"	2
23 SV	3	40,96	38,1	-	NI								
23 SV	5	35,93	33,69	-	4								
23 SV	6	37,56	33,62	-	2								
23 SV	7	37,41	-	-	NI								
23 SV	9	-	-	-	-								
24 EE	1	37,97	32,96	-	NI	M	Ad	871	38	01.07.2017	49°3'55.973"	17°3'21.443"	2
24 EE	3	39,23	34,09	-	5								
24 EE	4	-	-	-	-								
24 EE	5	-	-	-	-								
24 EE	6	-	-	-	-								
24 EE	7	-	-	-	-								
24 EE	8	-	-	-	-								
24 EE	9	-	-	-	-								
24 EE	10	-	-	-	-								
25 TM	2	32,78	29,49	-	6	F	Ad	63	32	08.08.2017	49°13'36.030"	16°30'33.548"	2
25 TM	3	-	-	-	-								
25 TM	10	-	-	-	-								
26 TM	2	34,08	33,36	-	2	F	Ad	71	36	08.08.2017	49°13'3.392"	16°35'52.181"	1B
26 TM	3	33,98	33,08	-	2								
26 TM	4	-	-	-	-								
26 TM	6	-	-	-	-								
26 TM	10	-	-	-	-								
27 TM	2	-	-	-	-	F	Ad	90	34	04.06.2017	49°11'38.457"	15°49'45.003"	1B
27 TM	3	-	-	-	-								
27 TM	4	-	-	-	-								
27 TM	6	-	-	-	-								
27 TM	10	-	-	-	-								
28 TM	2	35,15	30,39	-	2	M	Ad	56	29	11.08.2017	49°17'15.635"	16°27'24.082"	2
28 TM	3	-	-	-									
28 TM	4	-	-	-									
28 TM	6	-	-	-									
28 TM	10	-	-	-									
29 TM	2	-	-	-	-	M	Ad	69	37	26.06.2017	48°58'41.647"	14°26'49.180"	1B
29 TM	3	-	-	-	-								
29 TM	4	-	-	-	-								
29 TM	6	-	-	-	-								
29 TM	10	-	-	-	-								
30 SV	1	25,89	22,59	-	1, 2	F	Ad	263	57	18.08.2017	49°12'34.2"	16°39'04.6"	1A
30 SV	3	29,96	27,66	-	2, 4								
30 SV	4	35,85	34,56	-	1, 4								
30 SV	5	32,39	29,49	-	1, 2								
30 SV	6	38,83	36,89	-	7								
30 SV	7	39,53	-	-	2								
30 SV	8	36,99	34,46	-	4								
30 SV	9	38,82	36,38	-	4								
30 SV	10	-	37,05	-	4								
31 SV	1	38,61	34,57	-	1	F	Ad	175	58	16.08.2017	49°12'33.600"	16°35'10.544"	1B
31 SV	3	-	-	-	-								
31 SV	4	-	-	-	-								
31 SV	5	-	-	-	-								
31 SV	6	-	-	-	-								
31 SV	7	-	-	-	-								
31 SV	8	-	-	-	-								
31 SV	9	-	-	-	-								
31 SV	10	-	-	-	-								

32 EE	1	36,98	31,52	-	NI	M	Ad	629	42	04.05.2017	49°1'10.9"	14°26'7.5"	1B
32 EE	3	33,51	30,28	-	1								
32 EE	4	-	36,88	-	1, 4								
32 EE	5	-	34,47	-	4								
32 EE	6	-	-	-	-								
32 EE	7	-	-	-	-								
32 EE	8	-	-	-	-								
32 EE	9	-	-	-	-								
32 EE	10	-	-	-	-								
33 TM	2	-	-	-	-	M	Ad	84	40	12.08.2017	49°13'3.392"	16°35'52.181"	2
33 TM	3	39,52	-	-	1, 2								
33 TM	4	-	-	-	-								
33 TM	6	-	-	-	-								
33 TM	10	-	-	-	-								
34 EE	1	39,78	-	-	NI	F	Ad	818	48	10.06.2017	49°17'40.774"	16°20'45.750"	1A
34 EE	3	-	40,05	-	1								
34 EE	4	-	-	-	-								
34 EE	5	-	-	-	-								
34 EE	6	-	-	-	-								
34 EE	7	-	-	-	-								
34 EE	8	-	-	-	-								
34 EE	9	-	-	-	-								
34 EE	10	-	-	-	-								
35 TM	2	35,48	32,77	-	6	M	Ad	90	32	10.05.2017	48°58'07.80"	14°28'05.82"	1A
35 TM	3	-	-	-	-								
35 TM	4	-	-	-	-								
35 TM	6	-	-	-	-								
35 TM	10	-	-	-	-								
36 TM	2	-	-	-	-	F	Ad	96	31	25.04.2017	48°58'6.525"	14°28'6.938"	2
36 TM	3	-	37,74	-	NI								
36 TM	4	-	-	-	-								
36 TM	6	-	-	-	-								
36 TM	10	-	-	-	-								
37 TM	2	34,77	30,7	-	6	F	Ad	75	29	17.08.2017	49°13'3.392"	16°35'52.181"	1A
37 TM	3	-	-	-	-								
37 TM	4	-	-	-	-								
37 TM	6	-	-	-	-								
37 TM	10	-	-	-	-								
38 TM	2	37,06	33,25	-	2, 6	M	Ad	83	32	17.05.2017	49°3'39.78"	14°24'39.471"	1B
38 TM	3	-	-	-									
38 TM	4	-	-	-									
38 TM	6	-	-	-									
38 TM	10	-	-	-									
39 SV	1	-	-	-	-	M	Ad	345	45	09.05.2017	49°04'34.0"	14°31'52.2"	1A
39 SV	3	-	-	-	-								
39 SV	4	-	-	-	-								
39 SV	5	-	-	-	-								
39 SV	6	-	-	-	-								
39 SV	7	-	-	-	-								
39 SV	8	-	-	-	-								
39 SV	9	-	-	-	-								
39 SV	10	-	-	-	-								
40 SV	1	31,4	29,48	-	4	M	Ad	344	59	31.03.2017	49°29'43.307"	16°39'47.701"	1A
40 SV	3	-	-	-	-								
40 SV	4	-	-	-	-								
40 SV	5	-	-	-	-								
40 SV	6	-	-	-	-								
40 SV	7	-	45,35	-	NI								
40 SV	8	-	-	-	-								
40 SV	9	-	-	-	-								
40 SV	10	-	-	-	-								
41 SV	1	-	37,73	-	4	M	Juv	72	39	01.04.2017	ARS Plzeň	ARS Plzeň	1A
41 SV	3	-	-	-	-								
41 SV	4	-	-	-	-								
41 SV	5	-	-	-	-								
41 SV	6	-	-	-	-								
41 SV	7	-	-	-	-								
41 SV	8	-	-	-	-								
41 SV	9	-	-	-	-								
41SV	10	-	-	-	-								
42 TM	2	36,25	33,91	-	6	F	Ad	69	33	22.08.2017	49°12'51.360"	16°38'14.775"	2
42 TM	3	-	-	-	-								
42 TM	4	-	-	-	-								
42 TM	6	-	-	-	-								
42 TM	10	-	-	-	-								
43 TM	2	34,93	29,94	-	2	M	Ad	73	32,5	10.09.2017	49°13'3.392"	16°35'52.181"	1A
43 TM	3	-	-	-	-								
43 TM	4	-	-	-	-								
43 TM	6	-	-	-	-								
43 TM	10	-	-	-	-								
44 TM	2	36,91	33,99	-	1	F	Ad	70	31	22.09.2017	49°14'20.805"	16°32'9.751"	1B
44 TM	3	-	-	-	-								
44 TM	4	-	-	-	-								
44 TM	6	-	-	-	-								
44 TM	10	-	-	-	-								
45 EE	1	27,31	24,22	-	2, 5	M	Ad	478	34	VIII 2017	ARS Liberec	ARS Liberec	1A
45 EE	3	37,52	35,19	-	NI								
45 EE	4	-	-	-	-								
45 EE	5	-	-	-	-								
45 EE	6	33,04	28,47	-	3								
45 EE	7	-	-	-	-								
45 EE	8	-	-	-	-								
45 EE	9	-	-	-	-								
45 EE	10	-	-	-	-								

46 EE	1	-	-	-	-	F	Juv	74	19	VIII 2017	ARS Liberec	ARS Liberec	1B
46 EE	3	-	-	-	-								
46 EE	4	-	-	-	-								
46 EE	5	-	-	-	-								
46 EE	6	-	36,78	-	2								
46 EE	7	-	-	-	-								
46 EE	8	-	-	-	-								
46 EE	9	-	-	-	-								
46 EE	10	-	-	-	-								
47 EE	1	29,36	-	-	5	F	Ad	533	36	28.08.2017	48°59'33.101"	14°32'9.072"	1B
47 EE	3	34,83	-	-	5								
47 EE	5	-	-	-	-								
47 EE	6	-	-	-	-								
47 EE	7	-	-	-	-								
47 EE	8	37,15	-	-	5								
47 EE	9	-	-	-	-								
48 EE	1	40,92	38,82	-	1, 2	M	Ad	1335	39	2017	48°58'23.181"	14°44'33.756"	1B
48 EE	3	37,22	35,15	-	1								
48 EE	4	-	-	-	-								
48 EE	5	-	-	-	-								
48 EE	6	-	-	-	-								
48 EE	7	-	-	-	-								
48 EE	8	-	-	-	-								
48 EE	9	-	-	-	-								
48 EE	10	-	-	-	-								
49 EE	1	35,94	33,17	-	NI	F	Juv	343	37	30.09.2017	49°45'16.224"	14°31'59.680"E	1B
49 EE	3	30,02	26,86	-	1								
49 EE	4	37,35	33,25	-	NI								
49 EE	5	38,51	33,97	-	NI								
49 EE	6	34,53	31,95	-	NI								
49 EE	7	-	-	-	-								
49 EE	8	28,04	24,45	-	1								
49 EE	9	32,7	29,06	-	1								
50 EE	1	-	-	-	-	M	Ad	718	39	14.08.2017	48°57'44.526"	14°26'16.067"	2
50 EE	3	-	36,72	-	2								
50 EE	5	-	-	-	-								
50 EE	8	-	35,06	-	2								
51 SV	1	32,34	28,85	-	4	F	Ad	256	46	30.08.2017	49°03'26.4"	14°26'15.5"	2
51 SV	3	38,71	36,24	-	NI								
51 SV	5	32,52	27,8	-	2								
52 SV	1	40,56	34,29	-	4	M	Ad	368	51	24.09.2017	48°55'05.220"	14°30'03.780"	1A
52 SV	3	40,11	-	-	NI								
52 SV	4	-	-	-	-								
52 SV	5	37,77	33,33	-	4								
52 SV	6	-	-	-	-								
52 SV	7	-	-	-	-								
52 SV	9	-	-	-	-								
53 SV	1	39,49	34,67	-	NI	M	Ad	206	49	30.09.2017	49°03'40.0800"	14°24'53.100"	2
53 SV	3	-	-	-	-								
53 SV	4	-	-	-	-								
53 SV	5	38,79	34,24	-	2								
53 SV	6	-	-	-	-								
53 SV	7	-	38,02	-	NI								
53 SV	8	-	-	-	-								
53 SV	9	-	-	-	-								
54 TM	2	-	-	-	-	M	Ad	79	34	12.04.2017	48°57'27"	14°28'12"	1B
54 TM	3	-	-	-	-								
54 TM	4	-	-	-	-								
56 EE	1	40	-	-	NI	F	Ad	1187	38	VIII 2017	ARS Liberec	ARS Liberec	1A
56 EE	3	-	-	-	-								
56 EE	4	-	-	-	-								
56 EE	5	42,07	36,13	-	3								
56 EE	6	-	-	-	-								
56 EE	7	-	-	-	-								
56 EE	9	-	-	-	-								
56 EE	10	-	-	-	-								
57 EE	1	-	-	-	-	M	Ad	1118	39	VIII 2017	ARS Liberec	ARS Liberec	1A
57 EE	3	-	-	-	-								
57 EE	4	-	-	-	-								
57 EE	5	-	-	-	-								
57 EE	6	-	-	-	-								
57 EE	7	-	-	-	-								
57 EE	8	-	-	-	-								
57 EE	9	-	-	-	-								
57 EE	10	-	-	-	-								
58 ER	1	-	-	-	-	M	Ad	1005	33	02.04.2017	50°3'6.797"	14°25'21.164"	1B
58 ER	3	-	-	-	-								
58 ER	4	-	-	-	-								
58 ER	5	-	-	-	-								
58 ER	6	-	35,27	-	NI								
58 ER	7	-	-	-	-								
58 ER	8	-	-	-	-								
58 ER	9	-	-	-	-								
59 TM	2	42,6	32,93	-	1, 2	F	Ad	59	34	2017	ARS Plzeň	ARS Plzeň	2
59 TM	3	-	-	-									
59 TM	6	-	35,36	-	NI								
59 TM	10	-	35,01	-	2								
60 TM	2	36,51	32,2	-	6	M	Ad	80	35	23.04.2017	48°51'07.6"	14°21'50.5"	2
60 TM	3	-	-	-									
61 SV	1	-	-	-	-	F	Ad	335	53	30.09.2017	49°15'18.493"	17°41'50.894"	1A
61 SV	3	-	-	-	-								
61 SV	4	-	-	-	-								
61 SV	5	-	-	-	-								
61 SV	6	-	-	-	-								
61 SV	7	-	-	-	-								
61 SV	8	-	-	-	-								
61 SV	9	-	-	-	-								
62 EE	1	37,51	31,08	-	3	M	Ad	772	38	VIII 2017	ARS Liberec	ARS Liberec	1A
62 EE	3	32,94	26,78	-	NI								
62 EE	4	-	36,75	-	NI								
62 EE	5	-	34,76	-	NI								
62 EE	6	-	-	-	-								
62 EE	7	-	36,35	-	NI								
62 EE	8	26,25	20,1	-	3								
62 EE	9	39,05	35,53	-	NI								
62 EE	10	-	-	-	-								
63 EE	1	-	-	-	-	M	Ad	728	37	16.04.2017	48°57'58.3"	14°27'42.8"	2
63 EE	3	-	-	-	-								
63 EE	5	-	-	-	-								
64 EE	1	-	-	-	-	?	Ad	cca 500	37	18.05.2017	48°56'17.8"	14°28'44.1"	2
64 EE	3	-	-	-	-								
65 EE	1	-	-	-	-	?	Ad	722	36	22.06.2017	48°59'36.21"	14°32'30.44"	2
65 EE	3	-	-	-	-								
65 EE	8	-	32,76	-	3								
65 EE	9	-	-	-	-								
66 EE	1	35,96	32,34	-	1	M	Ad	780	39	01.08.2017	49°45'16.224"	14°31'59.680"E	1A
66 EE	3	34,6	31,09	-	1								
66 EE	4	-	-	-	-								
66 EE	5	-	-	-	-								
66 EE	6	-	-	-	-								
66 EE	9	-	-	-	-								
66 EE	10	-	-	-	-								

67 TM	2	-	-	-	-	F	Ad	89	33	25.09.2017	48°57'59.940"	14°27'47.340"	1B
67 TM	3	-	-	-	-								
67 TM	6	-	-	-	-								
67 TM	10	-	-	-	-								
68 TM	2	38,24	32,71	-	6	F	Ad	79	32	06.05.2017	50°55´24.000"	11°34´59.400"	1A
68 TM	3	-	-	-	-								
68 TM	4	-	-	-	-								
68 TM	6	-	-	-	-								
69 TM	2	-	-	-	-	F	Ad	101	36	15.03.2017	50°1'6.806"	14°44'19.495"	1A
69 TM	3	-	-	-	-								
69 TM	4	-	-	-	-								
69 TM	6	-	35,15	-	NI								
69 TM	10	-	-	-	-								
70 TM	2	-	-	-	-	F	Ad	94	35	06.04.2017	50°1'6.806"	14°44'19.495"	1A
70 TM	3	-	-	-	-								
70 TM	4	-	-	-	-								
70 TM	6	-	-	-	-								
70 TM	10	-	-	-	-								
72 TM	2	-	37,37	-	2	M	Ad	82	36	10.07.2017	48°58'31.44"	14°30'04.56"	2
72 TM	3	-	-	-	-								
72 TM	10	-	-	-	-								
73 TM	2	39,26	32,59	-	6	M	Ad	76	31	VIII 2017	ARS Liberec	ARS Liberec	1A
73 TM	3	-	-	-	-								
73 TM	4	-	-	-	-								
73 TM	6	-	-	-	-								
73 TM	10	-	-	-	-								
74 TM	2	31,93	26,63	-	2	F	Ad	77	32	VIII 2017	ARS Liberec	ARS Liberec	1A
74 TM	3	-	-	-	-								
74 TM	4	-	-	-	-								
74 TM	6	-	-	-	-								
74 TM	10	41,62	35,49	-	6								
75 TM	2	37,17	30,86	-	2	F	Ad	75	34	VIII 2017	ARS Liberec	ARS Liberec	1A
75 TM	3	-	-	-	-								
75 TM	4	-	-	-	-								
75 TM	6	-	-	-	-								
75 TM	10	-	-	-	-								
76 TM	2	-	-	-	-	F	Juv	50	34	VIII 2017	ARS Liberec	ARS Liberec	1A
76 TM	3	-	-	-	-								
76 TM	6	-	-	-	-								
77 TM	2	37,76	31,07	-	2, 6	M	Ad	67	30	23.08.2017	49°53'35.484"	14°23'41.175"	2
78 TM	2	40	35,65	-	6	F	Ad	79	32	08.05.2017	49°56'30.12"	15°15'40.80"	2!
78 TM	3	-	-	-									
78 TM	6	-	-	-									
78 TM	10	40,18	-	-	NI								
80 EE	1	27,47	24,13	-	1	F	Ad	320	34	31.10.2017	49°13'28.362"	16°31'1.976"	1A
80 EE	3	30,28	26,22	-	1, 6								
80 EE	4	41,04	37,33	-	1,4								
80 EE	5	35,73	30,47	-	NI								
80 EE	6	36,14	33,06	-	NI								
80 EE	7	35,53	30,57	-	NI								
80 EE	8	28,99	22,39	-	1, 4								
80 EE	9	34,06	30,56	-	NI								
80 EE	10	41,41	35,81	-	4								
81 ER	1	29,69	25,03	-	1	M	Juv	273	34	04.11.2017	49°25'27.911"	16°21'5.776"	1A
81 ER	3	33,81	29,08	-	2, 4								
81 ER	5	35,93	32,11	-	4								
81 ER	6	37,05	34,61	-	4								
81 ER	7	38,14	35,04	-	1, 4								
81 ER	8	29,27	23,9	-	1, 2, 4								
81 ER	9	36,02	33,17	-	1								
81 ER	10	-	35,07	-	NI								
82 EE	1	-	-	-	-	M	Juv	243	33	20.11.2017	49°29'33.673"	16°39'44.611"	1A
82 EE	3	-	-	-	-								
82 EE	4	-	-	-	-								
82 EE	5	-	-	-	-								
82 EE	6	-	-	-	-								
82 EE	7	-	-	-	-								
82 EE	8	39,84	34,58	-	2								
82 EE	9	-	-	-	-								
82 EE	10	-	-	-	-								
83 EE	1	-	-	-	-	F	Juv	143	25	06.11.2017	ARS Brno	ARS Brno	2
83 EE	3	-	-	-	-								
83 EE	4	-	-	-	-								
83 EE	5	-	-	-	-								
83 EE	6	-	-	-	-								
83 EE	7	-	-	-	-								
83 EE	8	-	34,78	-	NI								
83 EE	9	-	-	-	-								
83 EE	10	-	-	-	-								
84 EE	1	-	-	-	-	F	Juv	123	21	VIII 2017	ARS Liberec	ARS Liberec	2
84 EE	3	-	-	-	-								
84 EE	4	-	-	-	-								
84 EE	5	-	-	-	-								
84 EE	6	-	-	-	-								
84 EE	7	-	-	-	-								
84 EE	8	-	34,59	-	3								
84 EE	9	-	-	-	-								
84 EE	10	-	-	-	-								
85 EE	1	27,29	21,8	-	NI	M	Juv	268	35	06.11.2017	ARS Brno	ARS Brno	1A
85 EE	3	32,17	28,09	-	1								
85 EE	4	-	-	-	-								
85 EE	5	32,97	29,94	-	1								
85 EE	6	38,27	35,03		NI								
85 EE	7	38,38	35,07	-	1								
85 EE	8	28,58	25,35	-	1								
85 EE	9	37,03	33,24	-	NI								
85 EE	10	37,16	33,57	-	6								
86 ER	1	37,48	32,21	-	NI	F	Juv	273	31	01.11.2017	49°11'24.795"	16°21'38.179"	1A
86 ER	3	37,07	31,92	-	1, 4								
86 ER	4	-	35,35	-	4								
86 ER	5	37,84	35,67	-	4								
86 ER	6	-	-	-	-								
86 ER	7	34,73	32,98	-	1, 4								
86 ER	8	29,57	25,31	-	1								
86 ER	9	35,27	33,34	-	4								
86 ER	10	-	36,79	-	4								
87 EE	1	29,04	24,13	-	5	F	Juv	307	35	23.11.2017	49°17'56.374"	16°32'17.087"	1A
87 EE	3	34,91	31,14	-	5								
87 EE	4	-	-	-	-								
87 EE	5	-	-	-	-								
87 EE	6	37,75	34,4	-	2								
87 EE	7	36,23	33,19	-	1								
87 EE	8	33,8	30,65	-	2, 4								
87 EE	9	37,33	34,55	-	1, 2, 4, 5								
87 EE	10	-	-	-	-								
88 EE	1	38,18	34,44	-	5	F	Ad	440	35	VIII 2017	ARS Liberec	ARS Liberec	1B
88 EE	3	-	-	-	-								
88 EE	4	-	-	-	-								
88 EE	5	-	-	-	-								
88 EE	6	-	-	-	-								
88 EE	7	-	-	-	-								
88 EE	8	-	-	-	-								
88 EE	9	-	-	-	-								
88 EE	10	-	-	-	-								

89 ER	1	-	-	-	-	F	Ad	1005	33	21.05.2017	50°3'0.998"	14°27'31.651"	1A
89 ER	3	-	-	-	-								
89 ER	4	-	-	-	-								
89 ER	5	-	-	-	-								
89 ER	6	-	-	-	-								
89 ER	7	-	-	-	-								
89 ER	8	-	-	-	-								
89 ER	9	-	-	-	-								
89 ER	10	-	-	-	-								
90 EE	1	-	-	-	-	M	Ad	808	39	31.05.2017	49°03'33.5"	14°25'36.4"	2
90 EE	3	34,76	32,16	-	NI								
90 EE	8	35,85	31	-	NI								
90 EE	9	-	-	-	-								
91 EE	1	-	35	-	NI	M	Ad	752	38	05.07.2017	ARS Plzeň	ARS Plzeň	1A
91 EE	3	-	-	-	-								
91 EE	4	-	-	-	-								
91 EE	5	-	-	-	-								
91 EE	6	-	-	-	-								
91 EE	7	-	-	-	-								
91 EE	8	-	-	-	-								
91 EE	9	-	-	-	-								
91 EE	10	-	-	-	-								
92 EE	1	30,43	26,84	-	1	M	Ad	461	38	23.06.2017	ARS Plzeň	ARS Plzeň	1B
92 EE	3	31,04	27,86	35,56	1, 2								
92 EE	4	37,77	35,32	-	4								
92 EE	5	40,8	35,6	34,54	NI								
92 EE	6	-	34,32	33,78	NI								
92 EE	7	35,91	32,23	-	1, 4								
92 EE	8	31,51	27,82	36,79	1								
92 EE	9	37,83	33,06	35,23	NI								
92 EE	10	37,93	34,45	35,81	4								
93 EE	1	38,65	34,95	-	NI	F	Ad	464	31	V 2017	ARS Plzeň	ARS Plzeň	1A
93 EE	3	-	-	-	-								
93 EE	4	-	-	-	-								
93 EE	5	-	-	-	-								
93 EE	6	-	-	-	-								
93 EE	7	-	-	-	-								
93 EE	8	-	-	-	-								
93 EE	9	-	-	-	-								
93 EE	10	-	-	-	-								
94 EE	1	-	35,53	-	1, 3	M	Ad	1070	37	18.05.2017	48°58'44.825"	14°26'54.742"	1A
94 EE	3	-	-	-	-								
94 EE	4	-	-	-	-								
94 EE	5	-	-	-	-								
94 EE	6	38,77	-	-	1								
94 EE	7	-	-	-	-								
94 EE	8	-	-	-	-								
94 EE	9	-	-	-	-								
94 EE	10	-	-	-	-								
95 EE	1	31,5	25,99	-	NI	M	Juv	221	30	VIII 2017	49°11'24.795"	16°21'38.179"	1A
95 EE	3	26,89	23,86	-	1								
95 EE	4	37,95	-	-	4								
95 EE	5	32,68	29,21	-	4								
95 EE	6	38,62	33,78	-	2, 4								
95 EE	7	38,53	-	-	NI								
95 EE	8	29,48	25,28	-	1								
95 EE	9	34,95	31,95	-	4								
95 EE	10	38,32	34,77	-	1								
96 EE	1	-	39,96	-	NI	?	Ad	714	36	05.07.2017	49°6'15.540"	16°45'29.954"	2!
97 TM	2	-	-	-	-	?	Juv	44	34	22.08.2017	49°13'28.362"	16°31'1.976"	1A
97 TM	3	40,48	-	-	1								
97 TM	6	-	-	-	-								
97 TM	10	-	-	-	-								
98 EE	1	37	32,53	-	NI	F	Ad	385	34	19.05.2017	48°58'44.825"	14°26'54.742"	2
98 EE	3	-	-	-	-								
98 EE	9	-	-	-	-								
99 EE	1	38,89	-	-	NI	F	Ad	973	35	17.06.2017	48°56'28.500"	14°21'32.040"	1B
99 EE	3	-	-	-	-								
99 EE	4	-	-	-	-								
99 EE	5	-	-	-	-								
99 EE	6	-	-	-	-								
99 EE	7	-	-	-	-								
99 EE	8	-	-	-	-								
99 EE	9	-	-	-	-								
99 EE	10	-	-	-	-								
100 TM	2	38,24	32,6	-	2	F	Ad	87	32	15.07.2017	49°16'15.158"	16°35'47.066"	1A
100 TM	3	-	-	-	-								
100 TM	4	-	-	-	-								
100 TM	6	-	-	-	-								
100 TM	10	-	-	-	-								
101 TM	2	34,46	30,1	-	2, 6	F	Ad	92	30	25.10.2017	49°13'2.515"	16°36'51.924"	1A
101 TM	3	-	-	-	-								
101 TM	4	-	-	-	-								
101 TM	6	-	-	-	-								
101 TM	10	-	-	-	-								
102 TM	2	-	-	-	-	F	Ad	83	33	19.07.2017	49°13'3.392"	16°35'52.181"	1B
102 TM	3	-	-	-	-								
102 TM	4	-	-	-	-								
102 TM	6	-	-	-	-								
102 TM	10	-	-	-	-								
103 TM	2	-	-	-	-	M	Ad	79	34	31.03.2017	49°03'26.4"	14°26'15.5"	1B
103 TM	3	-	-	-	-								
103 TM	4	-	-	-	-								
103 TM	6	-	-	-	-								
103 TM	10	-	-	-	-								
104 TM	2	-	-	-	-	F	Ad	105	30	12.04.2017	48°57'27"	14°28'12"	1A
104 TM	3	-	-	-	-								
104 TM	4	-	-	-	-								
104 TM	6	-	-	-	-								
104 TM	10	-	-	-	-								
106 TM	2	39,84	33,98	-	1	F	Ad	73	33	26.08.2017	49°13'3.392"	16°35'52.181"	1A
106 TM	3	-	-	-	-								
106 TM	4	-	-	-	-								
106 TM	6	-	-	-	-								
106 TM	10	-	-	-	-								
107 TM	2	37,66	31,32	-	6	M	Ad	83	32	13.05.2017	50°18'6.749"	14°18'49.200"	2
107 TM	3	-	39,97	-	NI								
107 TM	4	-	-	-	-								
107 TM	6	-	-	-	-								
107 TM	10	-	-	-	-								
108 TM	2	-	-	-	-	F	Ad	78	32	25.04.2017	48°58'6.525"	14°28'6.938"	1B
108 TM	3	-	-	-	-								
108 TM	4	-	-	-	-								
108 TM	6	-	-	-	-								
108 TM	10	-	-	-	-								
109 TM	2	-	-	-	-	M	Ad	58	35	14.08.2017	49°17'56.370"	16°32'17.080"	2
109 TM	3	-	-	-	-								
109 TM	4	-	-	-	-								
109 TM	6	-	-	-	-								
109 TM	10	-	-	-	-								
111 TM	2	35,39	30,57	-	6	M	Ad	85	32	13.05.2017	50°17'54.512"	14°18'47.346"	1B
111 TM	3	-	-	-	-								
111 TM	4	-	-	-	-								
111 TM	6	-	-	-	-								
111 TM	10	-	-	-	-								

112 EE	1	38,09	34,7	-	1	M	Ad	487	40	25.01.2017	49°12'25.061"	16°29'23.562"	1A
112 EE	3	-	-	-	-								
112 EE	4	-	-	-	-								
112 EE	5	-	-	-	-								
112 EE	6	-	-	-	-								
112 EE	7	-	-	-	-								
112 EE	8	-	-	-	-								
112 EE	9	-	-	-	-								
112 EE	10	39,82	-	-	-								
114 TM	2	-	39,94	-	NI	M	Ad	87	32	16.05.2017	48°58'47.972"	14°27'13.146"	2
114 TM	3	-	-	-	-								
114 TM	4	-	-	-	-								
114 TM	6	-	-	-	-								
114 TM	10	-	-	-	-								
116 TM	2	30,2	22,77	-	2	F	Ad	65	36	V 2017	ARS Plzeň	ARS Plzeň	1B
116 TM	3	-	-	-	-								
116 TM	4	-	-	-	-								
116 TM	6	40,91	34,55	-	NI								
116 TM	10	-	36,27	-	NI								
117 TM	2	-	33,47	-	3	M	Ad	90	32	03.05.2017	48°58'32.2"	14°30'02.0"	2!
117 TM	3	-	-	-	-								
117 TM	6	-	-	-	-								
117 TM	10	-	-	-	-								
118 TM	2	-	-	-	-	F	Ad	81	34	V 2017	ARS Plzeň	ARS Plzeň	2
118 TM	3	-	-	-	-								
118 TM	4	-	-	-	-								
118 TM	6	-	-	-	-								
118 TM	10	-	-	-	-								
119 EE	1	29,78	25,29	-	1	F	Juv	290	36	02.12.2017	ARS Brno	ARS Brno	2
119 EE	3	33,58	28,74	35,81	1								
119 EE	4	-	-	-	-								
119 EE	5	34,09	29,62	35	NI								
119 EE	6	36,49	33,54	35,76	1								
119 EE	7	36,31	31,29	-	5								
119 EE	8	28,72	23,68	-	1, 5								
119 EE	9	35,51	31,65	-	1								
119 EE	10	-	-	-	-								
120 EE	1	30,96	26,71	-	5	M	Ad	372	34	19.01.2018	ARS Brno	ARS Brno	1A
120 EE	3	36,05	30,91	-	1, 3								
120 EE	4	-	-	-	-								
120 EE	5	-	-	-	-								
120 EE	6	-	-	-	-								
120 EE	7	-	-	-	-								
120 EE	8	-	-	-	-								
120 EE	9	-	-	-	-								
120 EE	10	-	36,59	-	1								
121 EE	1	33,54	29,02	-	NI	F	Juv	258	31	XII 2017	ARS Brno	ARS Brno	1B
121 EE	3	-	35,13	-	NI								
121 EE	4	-	-	-	-								
121 EE	5	-	-	-	-								
121 EE	6	-	36	-	NI								
121 EE	7	-	-	-	-								
121 EE	8	37,5	33,34	-	4, 5								
121 EE	9	38,01	-	-	5								
121 EE	10	-	34,82	-	NI								
122 EE	1	29,58	24,7	-	1, 3	F	Ad	341	37	06.12.2017	ARS Brno	ARS Brno	1A
122 EE	3	33,85	25,58	-	1, 2								
122 EE	4	-	-	-	-								
122 EE	5	35,89	29,18	-	4								
122 EE	6	-	33,52	-	5								
122 EE	7	-	29,82	-	1								
122 EE	8	30,57	25,04	-	1, 2								
122 EE	9	-	33,86	-	NI								
122 EE	10	-	-	-	-								
123 SV	1	40,46	33,77	-	NI	M	Ad	337	42	14.04.2017	49°36'05.2"	14°34'54.3"	1B
123 SV	3	41,61	35,53	-	4								
123 SV	4	-	-	-	-								
123 SV	5	-	-	35,05	-								
123 SV	6	-	-	36,3	-								
123 SV	7	-	-	36,2	-								
123 SV	8	41,13	40,17	-	NI								
123 SV	9	-	-	-	-								
124 SV	1	-	35,03	-	NI	M	Juv	110	39	VIII 2017	ARS Plzeň	ARS Plzeň	1A
124 SV	3	-	-	-	-								
124 SV	4	-	-	-	-								
124 SV	5	-	-	-	-								
124 SV	6	-	-	-	-								
124 SV	7	-	-	-	-								
124 SV	8	38,98	36,46	-	NI								
124 SV	9	-	-	-	-								
124 SV	10	-	-	-	-								
125 SV	1	-	-	-	-	?	Ad	237	46	04.10.2017	50°4'20.118"	14°25'28.149"	2
125 SV	3	-	-	-	-								
125 SV	6	-	35,5	-	4								
126 EE	1	28,53	24,29	-	1	F	Juv	282	35	01.02.2018	ARS Brno	ARS Brno	1A
126 EE	3	33,06	29,52	-	NI								
126 EE	4	-	-	-	-								
126 EE	5	36,35	31,04	-	NI								
126 EE	6	37,66	33,75	-	NI								
126 EE	7	-	34,91	-	NI								
126 EE	8	32,2	28,03	-	NI								
126 EE	9	39,28	-	-	NI								
126 EE	10	-	-	-	-								
127 EE	1	32,95	25,95	-	NI	M	Ad	359	36	23.12.2017	ARS Brno	ARS Brno	1A
127 EE	3	32,13	28,26	-	2, 5								
127 EE	4	-	-	-	-								
127 EE	5	36,48	-	-	5								
127 EE	6	37,51	36,08	-	1								
127 EE	7	-	-	-	-								
127 EE	8	-	35,19	-	NI								
127 EE	9	-	-	-	-								
127 EE	10	-	-	-	-								
128 TM	2	37,6	31,9	-	1	M	Ad	99	34	15.11.2017	48°58'39.187"	14°26'53.039"	1A
128 TM	3	-	-	-	-								
128 TM	4	-	-	-	-								
128 TM	6	-	-	-	-								
128 TM	10	-	-	-	-								
129 EE	1	34	28,27	-	1	F	Ad	380	37	13.10.2017	48°57'43.030"	14°26'27.964"	2
129 EE	3	31,8	28,22	-	1								
129 EE	5	38,92	33,43	-	NI								
129 EE	7	-	46,21	-	NI								
129 EE	8	32,66	29,12	-	1								
129 EE	9	37,99	-	-	NI								
130 EE	1	-	-	-	-	F	Ad	386	36	11.12.2017	49°53'06.7"	15°23'05.6"	1B
130 EE	3	-	-	-	-								
130 EE	8	-	-	-	-								
131 EE	1	33,17	29,78	-	1	M	Ad	469	33	16.10.2017	48°58'36.874"	14°26'52.579"	2
131 EE	3	31,26	27,98	-	1								
131 EE	4	-	-	-	-								
131 EE	5	33,68	29,71	-	1								
131 EE	6	36,95	32,87	-	1								
131 EE	7	-	35,96	-	NI								
131 EE	8	31,65	27,7	-	1								
131 EE	9	33,09	28,54	-	1								
131 EE	10	39,55	35,78	-	NI								

132 EE	1	34,33	30,1	-		NI	F	Juv	253	31	Winter 2017/18	ARS Přerov	ARS Přerov	1A
132 EE	3	38,09	34,81	-		NI								
132 EE	4	-	-	-		-								
132 EE	5	-	-	-		-								
132 EE	6	-	-	-		-								
132 EE	7	-	-	-		-								
132 EE	8	39,53	35,5	-		NI								
132 EE	9	-	35,57	-		NI								
132 EE	10	-	-	-		-								
133 EE	1	31,62	27,57	-		NI	M	Juv	270	34	Winter 2017/18	ARS Přerov	ARS Přerov	1A
133 EE	3	35,69	31,47	-		4								
133 EE	4	-	-	-		-								
133 EE	5	-	35,18	-		NI								
133 EE	6	37,02	34,33	-		NI								
133 EE	7	36,18	33,94	-		NI								
133 EE	8	-	-	-		-								
133 EE	9	37,05	-	-		NI								
133 EE	10	-	-	-		-								
134 EE	1	38,09	-	-		1	F	Ad	438	36	Winter 2017/18	ARS Přerov	ARS Přerov	1A
134 EE	3	-	-	-		-								
134 EE	4	-	-	-		-								
134 EE	5	-	-	-		-								
134 EE	6	-	-	-		-								
134 EE	7	40,74	-	-		NI								
134 EE	8	-	34,98	-		NI								
134 EE	9	-	-	-		-								
134 EE	10	-	-	-		-								
135 EE	1	32,23	29,16	-		1	F	Juv	244	35	Winter 2017/18	ARS Přerov	ARS Přerov	1A
135 EE	3	31,91	28,91	-		1								
135 EE	4	-	-	-		-								
135 EE	5	-	-	-		-								
135 EE	6	-	-	-		-								
135 EE	7	-	-	-		-								
135 EE	8	-	-	-		-								
135 EE	9	-	-	-		-								
135 EE	10	-	-	-		-								
136 SV	1	-	35,26	-		NI	M	Ad	306	43	21.03.2018	49°17'16.038"	16°27'24.391"	1A
136 SV	3	-	-	-		-								
136 SV	4	-	-	-		-								
136 SV	5	-	-	-		-								
136 SV	6	-	-	-		-								
136 SV	7	-	-	-		-								
136 SV	8	43,47	35,81	-		4								
136 SV	9	-	-	-		-								
136 SV	10	-	-	-		-								
137 EE	1	-	36,77	-		NI	F	Juv	104	24	Winter 2017/18	ARS Vlašim	ARS Vlašim	1B
137 EE	3	-	-	-		-								
137 EE	4	-	-	-		-								
137 EE	5	-	-	-		-								
137 EE	6	-	-		34,92	-								
137 EE	7	-	-	-		-								
137 EE	8	-	-	-		-								
137 EE	9	-	-	-		-								
137 EE	10	-	-	-		-								
138 EE	1	-	-	-		-	F	Juv	149	26	Winter 2017/18	ARS Vlašim	ARS Vlašim	1B
138 EE	3	-	-	-		-								
138 EE	4	-	-	-		-								
138 EE	5	-	-	-		-								
138 EE	6	-	-	-		-								
138 EE	7	-	-	-		-								
138 EE	8	-	-	-		-								
138 EE	9	-	-	-		-								
138 EE	10	-	-	-		-								
139 EE	1	-	-	-		-	F	Juv	103	25	Winter 2017/18	ARS Vlašim	ARS Vlašim	1B
139 EE	3	-	-	-		-								
139 EE	4	-	-	-		-								
139 EE	5	-	-	-		-								
139 EE	6	-	-	-		-								
139 EE	7	-	-	-		-								
139 EE	8	-	-	-		-								
139 EE	9	-	-	-		-								
139 EE	10	-	-	-		-								
140 EE	1	35,49	26,72	-		3	F	Juv	184	30	Winter 2017/18	ARS Vlašim	ARS Vlašim	1B
140 EE	3	30,9	23,31	-		NI								
140 EE	4	35,75	30,08	-		3								
140 EE	5	31,63	26,11	-		3								
140 EE	6	-	32,1	-		NI								
140 EE	7	-	33,89	-		3								
140 EE	8	-	29,54	-		3, 4								
140 EE	9	-	33,2	-		3								
140 EE	10	34,93	29,55	-		1, 3, 4								
141 EE	1	-	35,61	-		3	F	Ad	620	42	Winter 2017/18	ARS Liberec	ARS Liberec	2
141 EE	3	-	-	-		-								
141 EE	4	-	-	-		-								
141 EE	5	-	-	-		-								
141 EE	6	-	-	-		-								
141 EE	7	-	35,35	-		NI								
141 EE	8	-	-	-		-								
141 EE	9	-	-	-		-								
141 EE	10	-	37,34	-		NI								
142 EE	1	26,01	23,08	-		1, 5	M	Juv	309	30	Winter 2017/18	ARS Liberec	ARS Liberec	1B
142 EE	3	30,98	26,87	-		1, 5								
142 EE	4	-	-	-		-								
142 EE	5	36,12	31,91	-		4, 7								
142 EE	6	38,83	34,43	-		1, 2, 3								
142 EE	7	34,29	29,37	-		1, 4, 7								
142 EE	8	27,59	22,25	-		1, 3, 4								
142 EE	9	32,53	28,45	-		2								
142 EE	10	37,19	33,97	-		1								
143 EE	1	31,74	28,79	-		1	M	Juv	189	36	Winter 2017/18	ARS Liberec	ARS Liberec	1A
143 EE	3	32,94	30,24	-		1								
143 EE	4	-	-	-		-								
143 EE	5	40,61	32,88	-		1, 4								
143 EE	6	-	35,03	-		NI								
143 EE	7	39,88	34,48	-		4, 7								
143 EE	9	36,51	32,86	-		1, 5								
143 EE	10	37,82	-	-		3								
144 EE	1	26,31	23,2	-		1	M	Ad	324	36	Winter 2017/18	ARS Liberec	ARS Liberec	1A
144 EE	3	39,45	35,77	-		NI								
144 EE	4	-	-	-		-								
144 EE	5	-	-	-		-								
144 EE	6	-	-	-		-								
144 EE	7	-	-	-		-								
144 EE	9	-	35,45	-		NI								
144 EE	10	40,13	34,87	-		1								
145 TM	2	42,78	33,28	-		NI	M	Ad	76	33	Winter 2017/18	ARS Liberec	ARS Liberec	1A
145 TM	3	-	38,5	-		NI								
145 TM	4	-	-	-		-								
145 TM	6	-	-	-		-								
145 TM	10	-	-	-		-								

146 SV	1	41,21	34,69	-		NI	F	Ad	373	51	16.04.2018	49°14'30.601"	16°29'59.581"	1A
146 SV	3	40,79	37,23	-		NI								
146 SV	4	-	-	38,22	-									
146 SV	5	-	-	35,59	-									
146 SV	6	-	-	-	-									
146 SV	7	-	-	36,1	-									
146 SV	8	-	-	-	-									
146 SV	9	-	-	-	-									
146 SV	10	-	-	-	-									
147 TM	2	-	35,42	-		NI	F	Ad	87	31	Winter 2017/18	48°57'37.2"	14°29'03.3"	2
147 TM	3	-	-	-	-									
147 TM	6	-	-	-	-									
147 TM	10	-	-	-	-									
148 TM	2	-	-	-	-		F	Ad	82	36	Winter 2017/18	ARS Liberec	ARS Liberec	2
148 TM	3	-	-	-	-									
148 TM	4	-	-	-	-									
148 TM	6	-	-	-	-									
148 TM	10	-	-	-	-									
150 TM	2	39,15	33,25	-		2	M	Ad	86	34	Winter 2017/18	ARS Liberec	ARS Liberec	1A
150 TM	3	-	-	-	-									
150 TM	4	-	-	-	-									
150 TM	6	-	-	-	-									
150 TM	10	-	-	-	-									
151 TM	2	-	-	-	-		M	Ad	79	35	10.03.2018	50°21'26.533"	15°45'13.491"	1B
151 TM	3	-	-	-	-									
151 TM	4	-	-	-	-									
151 TM	6	-	-	-	-									
151 TM	10	-	-	-	-									
152 TM	2	-	-	-			M	Ad	64	37	III 2018	ARS Jaroměř	ARS Jaroměř	2
152 TM	3	-	-	-	-									
152 TM	4	-	-	-	-									
152 TM	6	-	-	-	-									
152 TM	10	-	-	-	-									
153 ER	1	40,49	35,84	-		NI	F	Ad	439	34	04.05.2018	49°12'28.418"	16°34'55.727"	2
153 ER	3	-	-	-	-									
153 ER	7	-	-	-	-									
153 ER	8	-	-	-	-									
154 EE	1	39,14	34,63	-		NI	F	Juv	151	33	Winter 2017/18	ARS Jaroměř	ARS Jaroměř	1B
154 EE	3	-	-	-	-									
154 EE	4	-	-	-	-									
154 EE	5	-	-	-	-									
154 EE	6	-	-	-	-									
154 EE	7	-	-	-	-									
154 EE	8	39,97	36,15	-		NI								
154 EE	9	-	-	-	-									
154 EE	10	-	-	-	-									
155 ER	1	34,96	29,29	-		NI	F	Ad	324	31	Winter 2017/18	ARS Jaroměř	ARS Jaroměř	1B
155 ER	3	34,46	30,65	-		1, 2								
155 ER	4	-	-	-	-									
155 ER	5	-	-	-	-									
155 ER	6	-	-	-	-									
155 ER	7	-	-	-	-									
155 ER	8	-	-	-	-									
155 ER	9	-	-	-	-									
155 ER	10	39,1	35,53	-		4								
156 TM	2	-	-	-	-		F	Ad	99	34	10.03.2018	49°17'17.651"	16°27'27.790"	1A
156 TM	3	-	-	-	-									
156 TM	4	-	-	-	-									
156 TM	6	-	-	-	-									
156 TM	10	-	-	-	-									
157 TM	2	40,11	33,79	-		NI	M	Ad	87	34	08.05.2018	49°18'11.924"	16°31'52.843"	1B
157 TM	3	-	-	-	-									
157 TM	4	-	-	-	-									
157 TM	6	-	-	-	-									
157 TM	10	-	-	-	-									
158 TM	2	-	-	-	-		F	Ad	75	34	08.05.2018	49°18'10.916"	16°31'52.920"	2
158 TM	3	-	-	-	-									
158 TM	4	-	-	-	-									
158 TM	6	-	-	-	-									
158 TM	10	-	-	-	-									
159 EE	1	37,92	34,05	-		NI	M	Ad	292	35	Winter 2017/18	ARS Jaroměř	ARS Jaroměř	1B
159 EE	3	37,17	-	-		5								
159 EE	4	-	-	-	-									
159 EE	5	-	-	-	-									
159 EE	6	-	-	-	-									
159 EE	7	40,98	-	-		5								
159 EE	8	-	36,59	-		NI								
159 EE	9	-	-	-	-									
159 EE	10	40,9	-	-		NI								
160 ER	1	-	35,04	-		NI	F	Ad	391	33	28.01.2018	50°17'51.088"	15°50'37.245"	1B
160 ER	3	-	35,03	-		NI								
160 ER	4	-	-	-	-									
160 ER	5	-	-	-	-									
160 ER	6	-	-	-	-									
160 ER	7	-	-	-	-									
160 ER	8	-	-	-	-									
160 ER	9	-	-	-	-									
160 ER	10	-	-	-	-									
161 TM	2	-	-	-	-		F	Juv	64	36	26.04.2018	49°13'3.392"	16°35'52.181"	1A
161 TM	3	-	-	-	-									
161 TM	4	-	-	-	-									
161 TM	6	-	-	-	-									
161 TM	10	-	-	-	-									
162 SV	1	37,64	34,96	-		NI	M	Ad	274	52	26.08.2017	49°18'42.545"	14°9'27.233"	1A
162 SV	3	-	-	-	-									
162 SV	4	-	-	-	-									
162 SV	5	-	35	-		NI								
162 SV	6	-	-	-	-									
162 SV	7	-	-	-	-									
162 SV	8	-	-	35,76	-									
162 SV	9	-	-	-	-									
162 SV	10	-	-	-	-									
163 SV	1	35,83	31,74	-		NI	M	Ad	331	42	Winter 2017/18	ARS Jaroměř	ARS Jaroměř	1B
163 SV	3	-	-	-	-									
163 SV	4	-	-	-	-									
163 SV	5	-	-	-	-									
163 SV	6	-	-	-	-									
163 SV	7	-	-	-	-									
163 SV	8	-	-	-	-									
163 SV	9	-	-	-	-									
163 SV	10	-	-	-	-									
164 SV	1	-	35,01	-		NI	F	Juv	117	47	06.04.2018	50°22'26.292"	15°53'16.240"	1B
164 SV	3	-	-	-	-									
164 SV	4	-	-	-	-									
164 SV	5	-	-	-	-									
164 SV	6	-	-	-	-									
164 SV	7	-	-	-	-									
164 SV	8	-	-	-	-									
164 SV	9	-	-	-	-									
164 SV	10	-	-	-	-									

165 SV	1	-	36,74	-	NI	M	Juv	76	43	19.06.2018	ARS Jaroměř	ARS Jaroměř	1A
165 SV	3	-	-	-	-								
165 SV	4	-	-	-	-								
165 SV	5	-	-	-	-								
165 SV	6	-	-	-	-								
165 SV	7	-	-	-	-								
165 SV	8	-	-	-	-								
165 SV	9	-	-	-	-								
165 SV	10	-	-	-	-								
166 EE	1	-	34,15	-	1	M	Ad	624	39	19.05.2018	49°12'57.860"	16°36'8.101"	1B
166 EE	3	-	-	-	-								
166 EE	8	-	-	-	-								
166 EE	9	-	-	-	-								
167 EE	1	-	37,54	-	4	M	Ad	508	39	13.05.2018	49°20'8.269"	15°34'39.406"	2
167 EE	3	-	-	-	-								
168 TM	2	-	-	-	-	F	Ad	100	32	11.05.2018	49°17'18.457"	16°27'34.587"	1A
168 TM	3	-	-	-	-								
168 TM	4	-	-	-	-								
168 TM	6	-	-	-	-								
168 TM	10	-	-	-	-								
169 TM	2	40,09	34,63	-	NI	M	Ad	89	34	11.05.2018	49°17'15.635"	16°27'24.700"	1A
169 TM	3	-	-	-	-								
169 TM	4	-	-	-	-								
169 TM	6	-	-	-	-								
169 TM	10	-	-	-	-								
