# Supplementary material for: Hedgehogs, Squirrels, and Blackbirds as Sentinel Hosts for Active Surveillance of Borrelia miyamotoi and Borrelia burgdorferi Complex in Urban and Rural Environments
Source: Microorganisms. 2020 Nov 30;8(12):1908. doi: 10.3390/microorganisms8121908 (PMC7760222; doi:10.3390/microorganisms8121908)
Supplement: Supplementary file 1 [file microorganisms-08-01908-s001.zip › Supplementary data manuscript FINAL/Table S4 Borrelia burgdorferi s.l.-positive animals database.pdf]

Table S4: *Borrelia burgdorferi* s.l.-positive animals database

EE = *Erinaceus europaeus* , ER = *Erinaceus roumanicus* , SV = *Sciurus vulgaris* , TM = *Turdus merula*

animal	ear	skin	muscle	blood	lungs	liver	spleen	urinary bladder	kidney	brain	results
2 ER	1	-	1, 2, 4	-	-	-	-	2, 4	-	-	mix infection 1, 2, 4
4 ER	NI	-	0	0	0	0	0	0	0	0	not identified
5 ER	NI	-	0	0	0	0	0	0	0	0	not identified
6 TM	-	6	0	0	-	-	-	-	-	0	*B. valaisiana*
7 EE	1, 7	-	2,5	4, 7	0	1	0	1, 2	1	1	mix infection 1, 2, 4, 5, 7
8 ER	0	-	0	0	0	0	0	1, 2	0	0	mix infection 1, 2
10 TM	-	6	0	0	-	0	-	-	-	0	*B. valaisiana*
11 TM	-	0	0	-	-	0	-	-	-	2	*B. garinii*
12 SV	2	-	0	0	0	0	0	1, 2	0	0	mix infection 1, 2
14 ER	0	-	1, 4	0	0	0	0	4	0	0	mix infection 1, 4
17 EE	1	-	0	0	0	0	0	0	0	0	*B. afzelii*
18 TM	-	2	0	0	-	NI	-	-	-	0	*B. garinii*
20 TM	-	2	0	0	-	0	-	-	-	0	*B. garinii*
21 SV	0	-	0	0	0	0	0	NI	0	0	not identified
22 SV	4	-	NI	0	0	0	0	0	0	0	*B. burgdorferi* sensu stricto
23 SV	4	-	NI	-	4	2	NI	-	0	-	mix infection 2, 4
24 EE	NI	-	5	0	0	0	0	0	0	0	*B. spielmanii*
25 TM	-	6	0	-	-	-	-	-	-	0	*B. valaisiana*
26 TM	-	2	2	0	-	0	-	-	-	0	*B. garinii*
28 TM	-	2	0	0	-	0	-	-	-	0	*B. garinii*
30 SV	1, 2	-	2, 4	1, 4	1, 2	7	2	4	4	4	mix infection 1, 2, 4, 7
31 SV	1	-	0	0	0	0	0	0	0	0	*B. afzelii*
32 EE	NI	-	1	1, 4	4	0	0	0	0	0	mix infection 1, 4
33 TM	-	0	1, 2	0	-	0	-	-	-	0	mix infection 1, 2
34 EE	NI	-	1	0	0	0	0	0	0	0	*B. afzelii*
35 TM	-	6	0	0	-	0	-	-	-	0	*B. valaisiana*
36 TM	-	0	NI	0	-	0	-	-	-	0	not identified
37 TM	-	6	0	0	-	0	-	-	-	0	*B. valaisiana*
38 TM	-	2, 6	0	0	-	0	-	-	-	0	mix infection 2, 6
40 SV	4	-	0	0	0	0	NI	0	0	0	*B. burgdorferi* sensu stricto
41 SV	4	-	0	0	0	0	0	0	0	0	*B. burgdorferi* sensu stricto
42 TM	-	6	0	0	-	0	-	-	-	0	*B. valaisiana*
**43 TM**	-	2	0	0	-	0	-	-	-	0	*B. garinii*
44 TM	-	1	0	0	-	0	-	-	-	0	*B. afzelii*
45 EE	2, 5	-	NI	0	0	3	0	0	0	0	mix infection 2, 3, 5
46 EE	0	-	0	0	0	2	0	0	0	0	*B. garinii*
47 EE	5	-	5	0	0	0	0	5	0	0	*B. spielmanii*
48 EE	1, 2	-	1	0	0	0	0	0	0	0	mix infection 1, 2
49 EE	NI	-	1	NI	NI	NI	0	1	1	0	*B. afzelii*
50 EE	0	-	2	-	0	-	-	2	-	-	*B. garinii*
51 SV	4	-	NI	-	2	-	-	-	-	-	mix infection 2, 4
52 SV	4	-	NI	0	4	0	0	-	0	-	*B. burgdorferi* sensu stricto
53 SV	NI	-	0	0	2	0	NI	0	0	-	*B. garinii*
56 EE	NI	-	0	0	3	0	0	-	0	0	*B. bavariensis*
58 ER	0	-	0	0	0	NI	0	0	0	-	not identified
59 TM	-	1, 2	0	-	-	NI	-	-	-	2	mix infection 1, 2
60 TM	-	6	0	-	-	-	-	-	-	-	*B. valaisiana*
62 EE	3	-	NI	NI	NI	0	NI	3	NI	0	*B. bavariensis*
65 EE	0	-	0	-	-	-	-	3	0	-	*B. bavariensis*
66 EE	1	-	1	0	0	0	-	-	0	0	*B. afzelii*
68 TM	-	6	0	0	-	0	-	-	-	-	*B. valaisiana*
69 TM	-	0	0	0	-	NI	-	-	-	0	not identified
72 TM	-	2	0	-	-	-	-	-	-	0	*B. garinii*
73 TM	-	6	0	0	-	0	-	-	-	0	*B. valaisiana*
74 TM	-	2	0	0	-	0	-	-	-	6	mix infection 2, 6
75 TM	-	2	0	0	-	0	-	-	-	0	*B. garinii*
77 TM	-	2, 6	-	-	-	-	-	-	-	-	mix infection 2, 6
78 TM	-	6	0	-	-	0	-	-	-	NI	*B. valaisiana*
80 EE	1	-	1, 6	1,4	NI	NI	NI	1, 4	NI	4	mix infection 1, 4, 6
81 ER	1	-	2, 4	-	4	4	1, 4	1, 2, 4	1	NI	mix infection 1, 2, 4
82 EE	0	-	0	0	0	0	0	2	0	0	*B. garinii*
83 EE	0	-	0	0	0	0	0	NI	0	0	not identified
84 EE	0	-	0	0	0	0	0	3	0	0	*B. bavariensis*
85 EE	NI	-	1	0	1	NI	1	1	NI	6	mix infection 1, 6
86 ER	NI	-	1, 4	4	4	0	1, 4	1	4	4	mix infection 1, 4
87 EE	5	-	5	0	0	2	1	2, 4	1, 2, 4, 5	0	mix infection 1, 2, 4, 5
88 EE	5	-	0	0	0	0	0	0	0	0	*B. spielmanii*
90 EE	0	-	NI	-	-	-	-	NI	0	-	not identified
91 EE	NI	-	0	0	0	0	0	0	0	0	not identified
92 EE	1	-	1, 2	4	NI	NI	1, 4	1	NI	4	mix infection 1, 2, 4
93 EE	NI	-	0	0	0	0	0	0	0	0	not identified
94 EE	1, 3	-	0	0	0	1	0	0	0	0	mix infection 1, 3
95 EE	NI	-	1	4	4	2, 4	NI	1	4	1	mix infection 1, 2, 4
96 EE	NI	-	-	-	-	-	-	-	-	-	not identified
97 TM	-	0	1	-	-	0	-	-	-	0	*B. afzelii*
98 EE	NI	-	0	-	-	-	-	-	0	-	not identified
99 EE	NI	-	-	-	-	-	-	-	-	-	not identified
100 TM	-	2	0	0	-	0	-	-	-	0	*B. garinii*
101 TM	-	2, 6	0	0	-	0	-	-	-	0	mix infection 2, 6
106 TM	-	1	0	0	-	0	-	-	-	0	*B. afzelii*
107 TM	-	6	NI	0	-	0	-	-	-	0	*B. valaisiana*
111 TM	-	6	0	0	-	0	-	-	-	0	*B. valaisiana*
112 EE	1	-	0	0	0	0	0	0	0	NI	*B. afzelii*
114 TM	-	NI	0	0	-	0	-	-	-	0	not identified
116 TM	-	2	0	0	-	NI	-	-	-	NI	*B. garinii*
117 TM	-	3	0	-	-	0	-	-	-	0	*B. bavariensis*
119 EE	1	-	1	0	NI	1	5	1, 5	1	0	mix infection 1, 5
120 EE	5	-	1, 3	0	0	0	0	0	0	1	mix infection 1, 3, 5
121 EE	NI	-	NI	0	0	NI	0	4, 5	5	NI	mix infection 4, 5
122 EE	1, 3	-	1, 2	0	4	5	1	1, 2	NI	0	mix infection 1, 2, 3, 4, 5
123 SV	NI	-	4	0	0	0	0	NI	0	-	*B. burgdorferi* sensu stricto
124 SV	NI	-	0	0	0	0	0	NI	0	0	not identified
125 SV	0	-	0	-	-	4	-	-	-	-	*B. burgdorferi* sensu stricto
126 EE	1	-	NI	0	NI	NI	NI	NI	NI	0	*B. afzelii*
127 EE	NI	-	2, 5	0	5	1	0	NI	0	0	mix infection 1, 2, 5
128 TM	-	1	0	0	-	0	-	-	-	0	*B. afzelii*
129 EE	1	-	1	-	NI	-	NI	1	NI	-	*B. afzelii*
131 EE	1	-	1	0	1	1	NI	1	1	NI	*B. afzelii*
132 EE	NI	-	NI	0	0	0	0	NI	NI	0	not identified
133 EE	NI	-	4	0	NI	NI	NI	0	NI	0	*B. burgdorferi* sensu stricto
134 EE	1	-	0	0	0	0	NI	NI	0	0	*B. afzelii*
135 EE	1	-	1	0	0	0	0	0	0	0	*B. afzelii*
136 SV	NI	-	0	0	0	0	0	4	0	0	*B. burgdorferi* sensu stricto
137 EE	NI	-	0	0	0	0	0	0	0	0	not identified
140 EE	3	-	NI	3	3	NI	3	3, 4	3	1, 3, 4	mix infection 1, 3, 4
141 EE	3	-	0	0	0	0	NI	0	0	NI	*B. bavariensis*
142 EE	1, 5	-	1, 5	0	4, 7	1, 2, 3	1, 4, 7	1, 3, 4	2	1	mix infection 1, 2, 3, 4, 5, 7
143 EE	1	-	1	0	1, 4	NI	4, 7	-	1, 5	3	mix infection 1, 3, 4, 5, 7
144 EE	1	-	NI	0	0	0	0	-	NI	1	*B. afzelii*
145 TM	-	NI	NI	0	-	0	-	-	-	0	not identified
146 SV	NI	-	NI	0	0	0	0	0	0	0	not identified
147 TM	-	NI	0	-	-	0	-	-	-	0	not identified
150 TM	-	2	0	0	-	0	-	-	-	0	*B. garinii*
153 ER	NI	-	0	-	-	-	0	0	-	-	not identified
154 EE	NI	-	0	0	0	0	0	NI	0	0	not identified
155 ER	NI	-	1, 2	0	0	0	0	0	0	4	mix infection 1, 2, 4
157 TM	-	NI	0	0	-	0	-	-	-	0	not identified
159 EE	NI	-	5	0	0	0	5	NI	0	NI	*B. spielmanii*
160 ER	NI	-	NI	0	0	0	0	0	0	0	not identified
162 SV	NI	-	0	0	NI	0	0	0	0	0	not identified
163 SV	NI	-	0	0	0	0	0	0	0	0	not identified
164 SV	NI	-	0	0	0	0	0	0	0	0	not identified
165 SV	NI	-	0	0	0	0	0	0	0	0	not identified
166 EE	1	-	0	-	-	-	-	0	0	-	*B. afzelii*
167 EE	4	-	0	-	-	-	-	-	-	-	*B. burgdorferi* sensu stricto
169 TM	-	NI	0	0	-	0	-	-	-	0	not identified

1 = *B. afzelii*
2 = *B . garinii*
3 = *B. bavariensis*
4 = *B. burgdorferi s.s.*
5 = *B. spielmanii*
6 = *B. valaisiana*
7 = *B. carolinensis/bissettii*
- tissue not obtained
0 = negative qPCR result
NI = positive qPCR, but genospecies not identifed
