# Supplementary material for: Hedgehogs, Squirrels, and Blackbirds as Sentinel Hosts for Active Surveillance of Borrelia miyamotoi and Borrelia burgdorferi Complex in Urban and Rural Environments
Source: Microorganisms. 2020 Nov 30;8(12):1908. doi: 10.3390/microorganisms8121908 (PMC7760222; doi:10.3390/microorganisms8121908)
Supplement: Supplementary file 1 [file microorganisms-08-01908-s001.zip › Supplementary data manuscript FINAL/Table S5 Erinaceus europaeus – combinations of Borrelia burgdorferi s.l. genospecies found in co-infected animals.pdf]

Table S5: *Erinaceus europaeus* – combinations of *Borrelia burgdorferi* s.l. genospecies found in animals with mixed infections

**Number of specimens**	***B. afzelii***	***B. garinii***	***B. bavariensis***	***B. burgdorferi s.s***	***B. spielmanii***	***B. valaisiana***	***B. bissettii /carolinensis***
1	X	X					
1	X		X				
1	X			X			
1	X				X		
1	X					X	
1				X	X		
**2**	X	X		X			
1	X	X			X		
1	X		X		X		
1	X			X		X	
1		X	X		X		
1	X		X	X			
1	X	X		X	X		
1	X	X	X	X	X		
1	X		X	X	X		X
1	X	X		X	X		X
1	X	X	X	X	X		X
