# Supplementary material for: Hedgehogs, Squirrels, and Blackbirds as Sentinel Hosts for Active Surveillance of Borrelia miyamotoi and Borrelia burgdorferi Complex in Urban and Rural Environments
Source: Microorganisms. 2020 Nov 30;8(12):1908. doi: 10.3390/microorganisms8121908 (PMC7760222; doi:10.3390/microorganisms8121908)
Supplement: Supplementary file 1 [file microorganisms-08-01908-s001.zip › Supplementary data manuscript FINAL/Table S8 Turdus merula – combinations of Borrelia burgdorferi s.l. genospecies found in co-infected animals.pdf]

Table S8: *Turdus merula* – combinations of *Borrelia burgdorferi* s.l. genospecies found in animals with mixed infections

Number of specimens	*B. afzelii*	*B. garinii*	*B. valaisiana*
2	X	X	
4		X	X
